# Anisotropic Properties of Epitaxial Ferroelectric Lead-Free 0.5[Ba(Ti_0.8_Zr_0.2_)O_3_]-0.5(Ba_0.7_Ca_0.3_)TiO_3_ Films

**DOI:** 10.3390/ma16206671

**Published:** 2023-10-13

**Authors:** Nicholas Cucciniello, Alessandro R. Mazza, Pinku Roy, Sundar Kunwar, Di Zhang, Henry Y. Feng, Katrina Arsky, Aiping Chen, Quanxi Jia

**Affiliations:** 1 Department of Materials Design & Innovation, University at Buffalo, The State University of New York, Buffalo, NY 14260, USA or ngcuccin@buffalo.edu (N.C.); henryfeng1@berkeley.edu (H.Y.F.); 2 Center for Integrated Nanotechnologies (CINT), Los Alamos National Laboratory, Los Alamos, NM 87545, USA; armazza@lanl.gov (A.R.M.); pinkur@lanl.gov (P.R.); sundar@lanl.gov (S.K.); dizhang@lanl.gov (D.Z.); 3 Department of Materials Science & Engineering, University of Illinois Urbana, Urbana, IL 61801, USA

**Keywords:** ferroelectric, anisotropy, dielectric, energy storage, morphotropic phase boundary

## Abstract

As the energy demand is expected to double over the next 30 years, there has been a major initiative towards advancing the technology of both energy harvesting and storage for renewable energy. In this work, we explore a subset class of dielectrics for energy storage since ferroelectrics offer a unique combination of characteristics needed for energy storage devices. We investigate ferroelectric lead-free 0.5[Ba(Ti_0.8_Zr_0.2_)O_3_]-0.5(Ba_0.7_Ca_0.3_)TiO_3_ epitaxial thin films with different crystallographic orientations grown by pulsed laser deposition. We focus our attention on the influence of the crystallographic orientation on the microstructure, ferroelectric, and dielectric properties. Our results indicate an enhancement of the polarization and strong anisotropy in the dielectric response for the (001)-oriented film. The enhanced ferroelectric, energy storage, and dielectric properties of the (001)-oriented film is explained by the coexistence of orthorhombic-tetragonal phase, where the disordered local structure is in its free energy minimum.

## 1. Introduction

Ferroelectric materials based on lead zirconate titanate Pb(Zr_1-x_Ti_x_)O_3_ (PZT) have found extensive applications within the modern electronics industry, namely actuators, sensors, and various energy storage and harvesting devices [1,2,3]. PZT exhibits superior dielectric and piezoelectric properties. However, such a chemical composition contains more than 60 wt.% lead (Pb). As a result of the high volatility and toxicity of Pb, it raises much concern about environmental contamination during the production and disposal of PZT materials. In recent years, there has been renewed interest in developing lead-free ferroelectric materials as restrictions on the use of Pb-based materials become more stringent globally [4,5,6]. This has led to a new push in materials research, with major focus on BaTiO_3_-based ferroelectrics [7]. Limited experimental results have shown that BaTiO_3_ (BTO)-based ceramics and thin films are very promising alternatives for Pb-based systems in electromechanical device applications. Numerous Ca-, Sr-, and Zr-modified BTO materials have been developed, including the Ba(Ti_0.8_Zr_0.2_)-x(Ba_0.7_Ca_0.3_)TiO_3_ (BZT-*x*BCT) crystal system [8]. Liu et al. found that a morphotropic phase boundary (MPB) exists at *x* = 0.5 for the BZT-*x*BCT system, which gives rise to a large piezoelectric response (d_33_ = ~620 pCN^−1^) [8]. At present, to increase the ferroelectric, dielectric, and piezoelectric properties of BZT-*x*BCT, most of the research has focused on composition tuning around or near the MPB to enhance properties similar to that of Pb-based systems [9,10,11]. Alternatively, through the introduction of an LaNiO_3_ buffer layer between the BZT-BCT thin film and the Pt/Ti/SiO_2_/Si substrate, Li et al. showed that the buffer layer could lead to better crystallinity and piezoelectric response due to the promotion of nucleation sites during growth [12]. Furthermore, it is widely acknowledged that orientation-engineered ferroelectric films exhibit significant variations in performance owing to their inherent anisotropy. This inherent anisotropy holds substantial importance in the context of materials design for microelectronic devices. Numerous reports have explored the connection between ferroelectric, dielectric, and piezoelectric characteristics, and orientation in various types of ferroelectric thin films, including those based on PbZr_1-x_Ti_x_O_3_, K_0.5_N_0.5_NbO_3_, BiFeO_3_, and Ba_1-x_Sr_x_TiO_3_ [13,14,15,16].

To date, limited research exists on the systematic investigation of the effects of different crystallographic orientations on the physical properties of BZT-*x*BCT. At the MPB, the anisotropic nature of BZT-*x*BCT arises from the crystal structure and arrangement of its constituent atoms. In relaxor ferroelectrics, the atomic arrangement is not perfectly ordered, resulting in microstructural frustrations within the crystal lattice. These localized regions encompass varying degrees of polarization direction with respect to the normal direction of the film, in particular, epitaxial films. These regions, known as polar nanoregions (PNRs), can contribute to the distinctive relaxor behavior largely in part due to the disordered state and the local structural fluctuations, where the polarization direction varies randomly on the microscopic scale [17]. The presence of PNRs in the nanoscale regions can lead to enhanced ferroelectric, dielectric, and piezoelectric properties. The disordered distribution of dipoles and ease of the dipoles to reorient once under an applied electric field make this relaxor-like behavior advantageous for fast-switching applications and energy storage devices as compared to nominal ferroelectrics that exhibit well-defined and highly organized ferroelectric domains possessing a high remanent polarization and large coercivity.

Here we report the structural, ferroelectric, and dielectric properties of epitaxial 0.5BZT-0.5BCT (or BZT-BCT for simplicity of future discussion) thin films grown on single crystal (001)-, (110)-, and (111)-oriented SrTiO_3_ substrates via pulsed laser deposition, where epitaxial SrRuO_3_ is used as a bottom electrode. The microstructures, ferroelectric, and frequency-dependent dielectric properties of the BZT-BCT thin films were investigated in relation to the respective crystallographic orientations. The coexistence of orthorhombic-tetragonal phases was identified in the (001)-oriented thin films, whereas the (110)- and (111)-oriented BZT-BCT films exhibited a single tetragonal phase. A large anisotropy exists in the frequency-dependent dielectric and tunability characteristics, suggesting the improved functional properties of the (001)-oriented film originate from fast domain switching and polarization rotation arising from the diminished energy barrier. The observation of the anisotropic properties of BZT-BCT thin films introduced by the crystallographic orientations of the substrates may open other possibilities for designing high-performance energy storage devices.

## 2. Results

### 2.1. Structural Analysis

**Figure 1:**
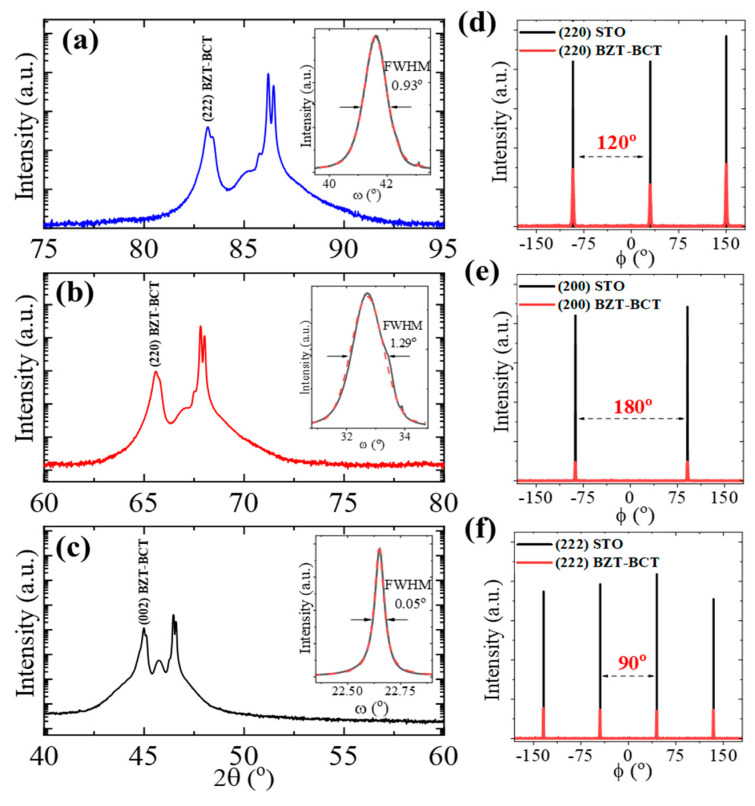
X-ray diffraction 2θ-scans of the BZT-BCT films on SRO-buffered (**a**) (111), (**b**) (110), (**c**) (001) STO substrates, where the unlabeled diffraction peaks are from the SRO and STO. Insets of (**a**–**c**) present the rocking curve of (222), (220), and (002) diffractions of the BZT-BCT films on their corresponding substrates. (**d**) In-plane ϕ-scan of the (220) reflection of the BZT-BCT thin film on (111) STO. (**e**) In-plane ϕ-scan of the (200) reflection of the BZT-BCT thin film on (110) STO. (**f**) In-plane ϕ-scan of the (222) reflection of the BZT-BCT thin film on (001) STO.

Figure 1a–c show X-ray diffraction (XRD) 2θ-ꞷ scan patterns of the BZT-BCT films on SrRuO_3_ (SRO)-buffered SrTiO_3_ (STO) substrates with different crystallographic orientations of the substrates. Well-defined reflections corresponding to the (001)-, (110)-, and (111)-oriented films, matching that of the corresponding substrate orientation, suggest the growth of highly oriented BZT-BCT films (see the section on Materials and Methods). The formation of pure BZT-BCT films on different oriented substrates can be attributed to the small lattice mismatch between the film and the underlying SRO/STO substrate. It is noted that SRO serves two purposes. First, it acts as a buffer layer to control the lattice-induced strain for epitaxial growth of BZT-BCT (lattice parameter ~4.002 Å). This is made evident by considering SRO as pseudo-cubic with a lattice constant ranging between 3.941 Å–3.956 Å, in comparison with STO with a lattice parameter of 3.905 Å. This method has been demonstrated by Park et al. [18], whereby inserting a very thin interlayer between the substrate and the main layer of epitaxial Ba_0.6_Sr_0.4_TiO_3_, the strain state could be systematically controlled. It has been also reported that such a buffer layer with a lattice parameter between the film and the substrate can play a critical role in film quality [19]. Secondly, SRO has high thermal stability and conductivity. It can be used as a quality bottom electrode for the capacitors [20]. The full-width at half-maximum (FWHM) of (222), (220), and (002) is 0.93°, 1.29°, and 0.05° for the BZT-BCT films on (111), (110), and (001) STO, respectively (inset Figure 1a–c). The extremely narrow rocking curve of the (001)-oriented film indicates a much-improved crystalline quality of the BZT-BCT film grown under our optimized processing conditions. The rocking curve FWHM difference between different orientations could be due to the different growth dynamics. To further explore the in-plane alignment of the BZT-BCT thin films with respect to the SRO/STO, in-plane ϕ-scan measurements are performed. Figure 1d–f show the XRD in-plane ϕ-scans around the (220), (200), and (222) reflections of the BZT-BCT films on (111), (110), and (001) STO substrates, respectively. Characteristics of three-fold (120° apart), two-fold (180° apart), and four-fold (90° apart) symmetry of the film on STO substrates with different substrate orientations are understandable by considering the shape of the basal-plane of the STO substrates with different crystallographic orientations.

Based on the XRD 2θ-ω and ϕ-scans, the epitaxial relationship between the film and the substrate can be described as (111)_BZT-BCT_ || (111)_STO_ (and <220>_BZT-BCT_ || <220>_STO_), (110)_BZT-BCT_ || (110)_STO_ (and <002>_BZT-BCT_ || <002>_STO_), and (001)_BZT-BCT_ || (001)_STO_ (and <222>_BZT-BCT_ || <222>_STO_) for the BZT-BCT films on (111)-, (110)-, and (001)-oriented STO substrates, respectively.

**Figure 2:**
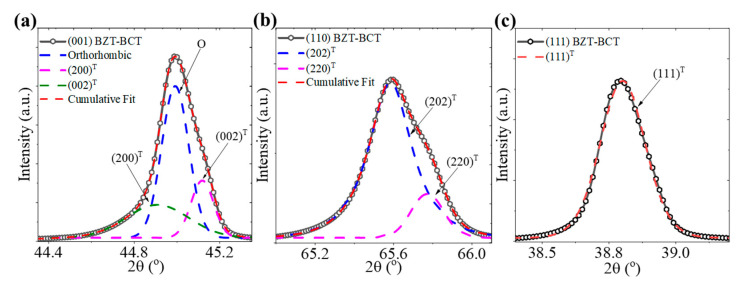
Enlarged view of the diffraction peaks and the resulting deconvolution and fitting of the (**a**) (002), (**b**) (220), and (**c**) (111) reflections.

It has been reported that BZT-BCT shows distinctive peak splitting along the (200) and (220), transforming into (002)/(200) and (202)/(220) doublets. Such an observation has been attributed to the phase shift to a tetragonal (T) phase structure [21,22]. To further investigate the phase structure of the epitaxial BZT-BCT films, Figure 2a–c shows the analysis of the corresponding deconvolution and peak fitting around the (002) at 2θ = ~45.0°, (220) at 2θ = ~65.6°, and (111) at 2θ = ~38.8° of the epitaxial BZT-BCT films on differently oriented substrates. The asymmetric nature of the (002) and (220) peaks for the BZT-BCT films on the (001) and (110) substrates, respectively, and the symmetrical nature of the (111) peak for the BZT-BCT film on the (111) STO substrate suggest the formation of tetragonal (T) phase films. Interestingly, on the (001)-oriented BZT-BCT film exhibits a superposition of the doublet T phase along with a single intense peak that can be defined as an orthorhombic phase. The formation of mixed phases of tetragonal and rhombohedral crystal structures has been reported for polycrystalline bulk-like ceramic samples [23,24].

It is noted that the coexistence of multiple phases in the BZT-BCT is still under intense study. For instance, some studies suggested BZT-BCT phase transitions from a low-temperature rhombohedral (R) phase to a room-temperature T phase at the MPB [8,11]. Recent work on the BZT-BCT phase diagram also indicated that an intermediate phase exists between the R and T phase transition [25]. High-resolution synchrotron X-ray characterization and more detailed studies of BZT-BCT have revealed that an MPB appears at the orthorhombic (O) to T phase transition [26,27]. At the MPB, the coexistence of the O + T phases results in enhanced ferroelectric and piezoelectric properties when stable and metastable phases exist, enabling direct domain and easy polarization switching. This is directly correlated to the minimum energy barrier in the mixed phase [28,29,30]. Our previous work has explored the microscopic mechanism of the relaxor-type behavior in BZT-BCT thin films. Our results have shown that the existence of high density nanodomains in BZT-BCT thin films and Sn doping further breaks down the domain size and reduces the polarization anisotropy [31].

### 2.2. Ferroelectric and Energy Storage Properties

**Figure 3:**
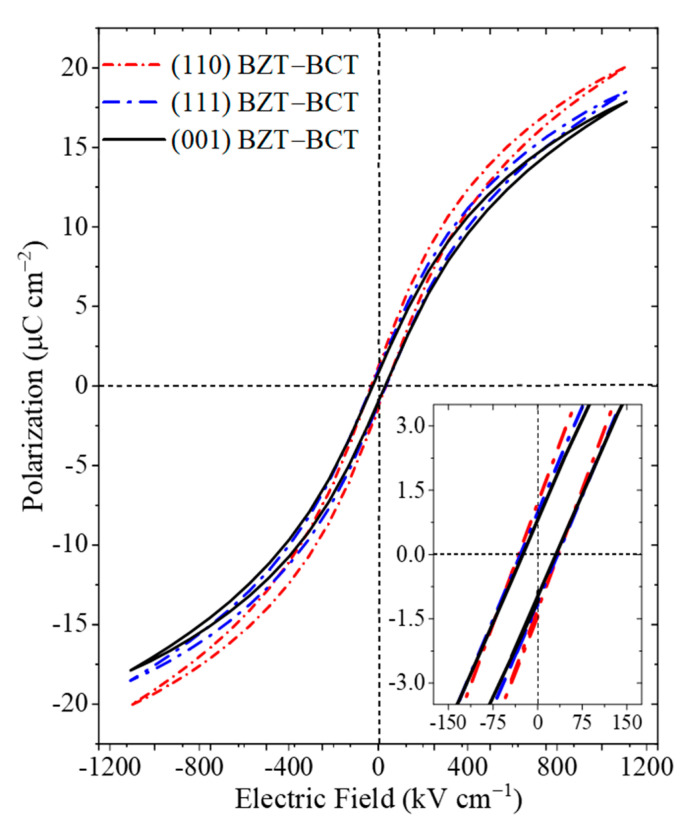
Polarization vs. electric field hysteresis loops for (001)-, (110)-, and (111)-oriented BZT-BCT films. The inset shows the magnification around the remanent polarization (*P_r_*) and coercive field (*E_c_*).

Figure 3 shows the room temperature polarization vs. electric field (*P-E*)-hysteresis loops for the (001)-, (110)-, and (111)-oriented BZT-BCT films, at a fixed frequency of 1 kHz. Well-defined slim *P-E* loops are present for all films, confirming typical ferroelectric behavior of the materials. Interestingly, we have noticed that there is a clear trend for the remanent polarization (*P_r_*) and coercive field (*E_c_*) when the BZT-BCT films are deposited on different oriented substrates, i.e., *P_r_*^(001)^ < *P_r_*^(111)^ < *P_r_*^(110)^ and *E_c_*^(001)^ < *E_c_*^(111)^ < *E_c_*^(110)^. The values of some important parameters related to the ferroelectric materials are listed in Table 1. The absolute values of *P_r_* and *E_c_* for our (001)-, (110)-, and (111)-oriented BZT-BCT films can be compared to the ferroelectric properties reported by two separate studies conducted by Lou et al., one being an orientation dependent study where the reported *P_r_* and *E_c_* for their (001)-, (110)-, and (111)-oriented BZT-BCT epitaxial thin films on La_0.7_Sr_0.3_MnO_3_-buffered STO substrates are 4.14 μC cm^−2^, 2.39 μC cm^−2^, and 1.67 μC cm^−2^ and 59.7 kV cm^−1^, 39.6 kV cm^−1^, and 60.2 kV cm^−1^, respectively [32]. Furthermore, in an independent study of (110)-oriented BZT-BCT on SRO-buffered STO, Lou et al. reported a *P_r_* of 2.01 μC cm^−2^ and an *E_c_* of 187 kV cm^−1^ [33]. The variation could be attributed to the difference in growth techniques, growth conditions, film thickness, substrate quality, and the bottom electrode materials.

The ferroelectric properties of the (001)-oriented BZT-BCT thin film differs from previous results based on the anisotropic properties of BTO-based films [34,35,36], as the energy barrier for polarization rotation is at the free energy minima from the phase coexistence seen in the (001)-oriented film. This may come from the reduction in the anisotropy energy in the BZT-BCT film with the coexistence of the O + T phase, which leads to the minimization of the orientation preferences of the PNRs, resulting in greater isotropic responses to the electric field as compared to the (110)- and (111)-oriented BZT-BCT films that exhibit a single T phase only.

**Figure 4:**
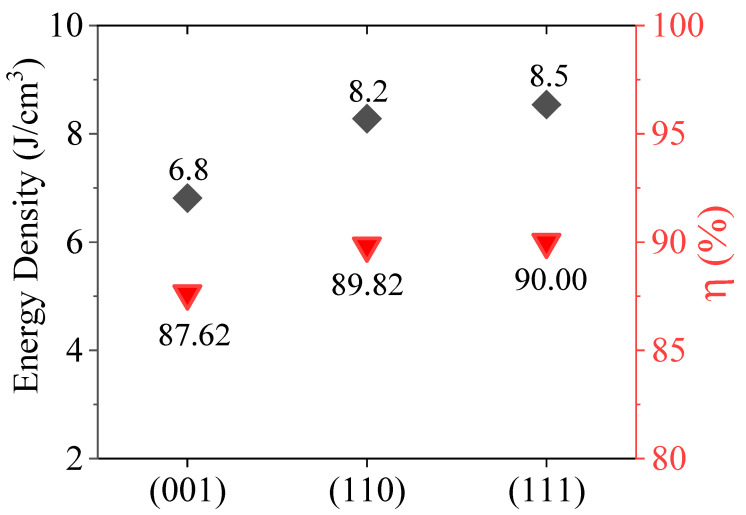
Energy storage density and efficiency of the (001)-, (110)-, and (111)-oriented BZT-BCT films.

To evaluate the effect of film orientation on the energy storage of the epitaxial BZT-BCT thin films with different crystallographic orientations, we calculated the energy density and efficiency (*η*) from the unipolar *P-E* loop based on the following equations:

(1) $$W_{\mathrm{rec}}=\int_{P_{r}}^{P_{max}}EdP$$

(2) $$\eta \;(\%)=\int_{P_{r}}^{P_{max}}EdP/\int_{0}^{P_{max}}EdP$$

where *W_rec_* is the recoverable energy storage density, *P_max_* is the maximum polarization, *P_r_* is the remanent polarization, and *E* is the electric field, respectively [37,38]. From the *P-E* loops shown in Figure 3, it is clear that the (001)-, (110)-, and (111)-oriented BZT-BCT films possess different *P_max_* and *P_r_* at an electric field of 1150 kV cm^−1^, which is lower than their breakdown field. Figure 4 summarizes the energy density and efficiency of the different orientated BZT-BCT films. The (111)-oriented BZT-BCT thin film exhibits an *η* of 90.00, while the (110) and (001) films show slightly lower values of 89.82 and 87.62%, respectively. The energy density and efficiency of recent reports on BZT-*x*BCT are listed in Table 2.

### 2.3. Dielectric and Tunability Properties

**Figure 5:**
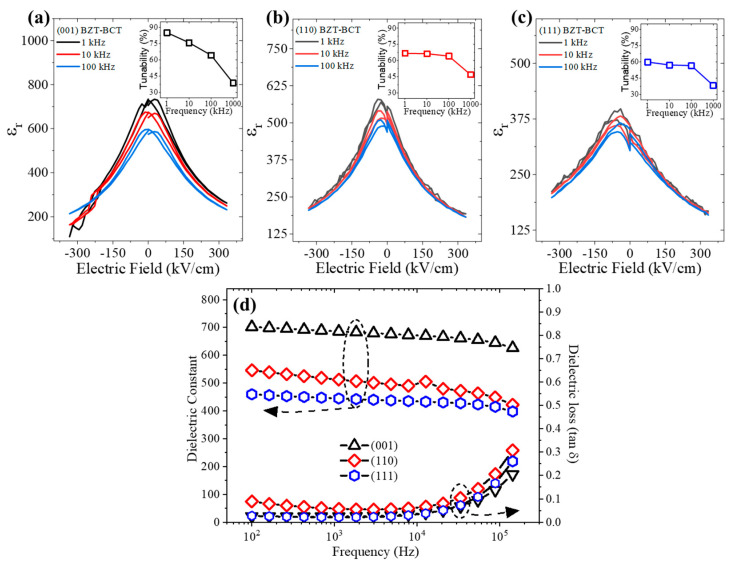
Dielectric constant vs. electric field at different frequencies of the (**a**) (001)-, (**b**) (110)-, and (**c**) (111)-oriented BZT-BCT thin films. The insets show the tunability (%) at a frequency of 1, 10, 100, and 1000 kHz. (**d**) Dielectric constant and dielectric loss as a function of frequency.

Figure 5a–c depict the dielectric constant (*ε_r_*) vs. the electric field (*E*) characteristics at different frequencies, where the DC field was swept from positive to negative and back to positive. To evaluate the dielectric constant, *ε_r_*, of the BZT-BCT films with different orientations, capacitance vs. voltage (*C-V*) measurements are taken at room temperature under a DC bias (330 kV cm^−1^) at frequencies of 1 kHz, 10 kHz, and 100 kHz. It is well known that ferroelectric materials typically show distinctive butterfly-shaped *ε_r_* vs. *E* loops, where the *ε_r_* is at its maximum at near zero field. The value of the dielectric constant decreases with increasing the field. The scaling of the dielectric response with the applied field agrees well with the domain process of ferroelectrics [42]. On the other hand, it is very interesting to notice that the maximum *ε_r_* of the BZT-BCT films is greatly affected by the orientation of the films. In other words, the BZT-BCT films show a large anisotropy in *ε_r_* with the relationship of *ε_r_*^(001)^ > *ε_r_*^(110)^ > *ε_r_*^(111)^. The absolute value of the *ε_r_* is 702, 546, and 459 at a frequency of 1 kHz for the (001)-, (110)-, and (111)-oriented BZT-BCT thin films, respectively. It should be emphasized that the *ε_r_* of the epitaxial BZT-BCT films is significantly lower than their bulk counterparts (*ε_r_* > 2400), potentially due to the effect of smaller grain size and substrate clamping [43,44]. Much smaller *ε_r_* of epitaxial films such as Ba_1-x_Sr_x_TiO_3_ in comparison with their bulk counterpart has also been reported, where the crystal structure distortion such as the lattice strain can also play an important role in the absolute value of *ε_r_* for the given material [45,46]. We would also like to point out that the field at the maximum *ε_r_* shifts slightly to the negative field direction for the (110)- and (111)-oriented films. This may be a result of the interface effects between the film and electrodes used in device fabrication.

The dielectric tunability (*DT*) was extracted from the *C-V* data and calculated using:

*DT* (%) = [*ε_r_*(0) − *ε_r_*(*E*)]/*ε_r_*(0) × 100 (3)

where *ε_r_*(0) and *ε_r_*(*E*) are the dielectric constant at zero field and a given applied field, respectively. The inset of Figure 5a–c summarizes the tunability as a function of frequency for the three oriented BZT-BCT films. As can be seen from Figure 5, the (001)-oriented BZT-BCT thin film exhibits a moderately larger tunability with a relationship of *DT*^(001)^ > *DT*^(110)^ > *DT*^(111)^ at a relatively lower frequency (<100 kHz). The tunability of the BZT-BCT films at 1 kHz is 85.09, 66.67, and 59.89% for the (001)-, (110)-, and (111)-oriented films, respectively. It is noted that the tunability of the BZT-BCT films with different orientations decreases with increasing frequency. At relatively higher frequencies (>100 kHz), the tunability seems to become similar, regardless of the orientation of the films. The frequency-dependent *ε_r_* and dielectric loss (tanδ) of the BZT-BCT films with different orientations are shown in Figure 5d. Throughout the entire frequency range, the *ε_r_* demonstrated a descending order of *ε_r_*^(001)^ > *ε_r_*^(110)^ > *ε_r_*^(111)^. The dielectric loss of the BZT-BCT films, on the other hand, is in the same range with no large difference, although the (110)-oriented BZT-BCT film shows a slightly larger dielectric loss. Similar behavior was also reported in other ferroelectric films, which has been attributed to the space charging effects, interfacial diffusion, and contact resistance [47,48,49]. It is also important to note that all the BZT-BCT films show a small frequency dispersion of *ε_r_* at frequencies from 100 Hz–100 kHz, which can be ascribed to the dynamics of the PNRs in the epitaxial BZT-BCT films as well as the permanent dipole moment retention [49].

## 3. Discussion

The polarization vector angle with respect to the surface normal changes with the crystallographic orientation direction for ferroelectric systems with tetragonal structure such as BaTiO_3_. In the majority of studies of ferroelectric materials, anisotropic properties originate in the <110> and <111> directions [50,51,52]. For instance, in a study of the orientation dependence of BaTiO_3_ thin films, Zhang et al. suggested that the further the tilting angle of the polar axis away from the crystallographic orientation, the greater the dielectric response becomes [34]. However, in the present study, we show that the superior dielectric response lies along the <001> direction. This is likely a result of phase coexistence (O + T) and tetragonal phase instability as described below.

Our work shows that (001)-oriented BZT-BCT films exhibit the coexistence of O + T phases, whereas the (110)- and/or (111)-oriented epitaxial BZT-BCT films do not. The enhancement of dielectric properties along certain crystallographic orientations could, for this reason, be explained by the local PNRs directly related to the microstructures, where the coexistence of different phases at room temperature and the specific composition of *x* = 0.5 can lead to a generally flattened energy barrier [53,54,55,56,57]. Under applied electric fields, the ease of domain switching and polarization rotation can come from the instability of the polarization direction at the MPB characterized by an ultra-low-energy barrier, leading to heightened ferroelectric and piezoelectric responses [58]. This is in contrast with the (110)- and (111)-oriented epitaxial films where the XRD analysis presents a single-phase T structure. Furthermore, the single T phase at room temperature results in smaller fluctuations of polarization as the T phase is stable. It is therefore that an increase in the barrier height in the free energy profile and anisotropy energy may result in different properties [28].

We note that although both (110)- and (111)-orientated epitaxial films show relatively a lower dielectric constant as a result of the formation of stable single tetragonal phase, the nonequilibrium process of pulsed laser deposition may also play a role in the dielectric properties of the films. As all BZT-BCT thin films were grown under the same processing conditions, the ultimate stoichiometry and microstructure of the thin films can change with respect to the crystallographic orientation, which may result in defects (i.e., dislocations, impurities, vacancies, etc.) and the formation of defect-dipole complexes [59]. Furthermore, the residual stress and lattice strain vary by orientation, where the polarization displacement of the heterovalent cations (Ba^2+^, Ca^2+^, Ti^4+^, Zr^4+^) with respect to the oxygen octahedra of perovskite films affect the preferentially oriented BZT-BCT films that are largely seen in the dielectric response.

## 4. Materials and Methods

Epitaxial BZT-BCT thin films with a thickness of ~270 nm were synthesized on 50 nm thick conducting SrRuO_3_ (SRO)-buffered SrTiO_3_ substrates with orientations of (001), (110), and (111) via pulsed laser deposition (KrF excimer laser, λ = 248 nm). The thickness of the epitaxial BZT-BCT films were calculated via the deposition rate and cross-sectional transmission electron microscopy. The processing conditions were optimized to achieve high-crystallinity films. Briefly, the substrate temperature and oxygen partial pressure were maintained at 687 °C and 50 mTorr, respectively, during film growth. A laser energy of 1.5 J cm^−2^ at 5 Hz was used to ablate the BZT-BCT target. In order to obtain a uniform laser energy density on the target, a rectangular-shaped laser spot, defined by the image beam technique [60], was used in our experimental setup. After deposition, the films were annealed (in situ) at 500 °C in 760 Torr oxygen for one hour before being cooled down to room temperature at a ramping rate of 5 °C min^−1^. To conduct dielectric property measurements, circular Au top electrodes (~100 nm thick) with a diameter of 350 μm defined by a shadow mask were then deposited by magnetron sputtering at room temperature. More detailed growth and optimization were reported elsewhere [31,61].

Capacitors with a configuration of Au/BZT-BCT/SRO/STO were characterized by measuring the polarization vs. electric field (*P-E*) hysteresis loops, and the capacitance vs. voltage (*C-V*) characteristics using a ferroelectric test system (Precision Premier II; Radiant Technologies, Inc., Albuquerque, NM, USA). The dielectric properties of the BZT-BCT films were further measured using a precision LCR meter (E4980, Keysight Agilent, Santa Rosa, CA, USA) at room temperature with a frequency range of 1 kHz–1 MHz. The crystallographic properties and microstructure of the films were analyzed via X-ray diffraction (XRD, Empyrean, Malvern PANalytical, Westborough, MA, USA).

## 5. Conclusions

In summary, epitaxial BZT-BCT thin films have been successfully grown on (001)-, (110)-, and (111)-oriented SRO-buffered STO substrates via pulsed laser deposition. XRD analysis reveals the coexistence of the orthorhombic and tetragonal phases at room temperature for the (001)-oriented film as compared to a single tetragonal phase for the (110)- and (111)-oriented films. The ferroelectric properties show typical ferroelectric behavior with all orientations exhibiting superior electrical energy efficiencies (*η* > 87%), suggesting such materials as quality candidates for potential energy storage applications. The dielectric constant and tunability extracted from *C-V* curves displays the existence of a large anisotropy between the (001)-, (110)-, and (111)-oriented epitaxial BZT-BCT films with a maximal *ε_r_* and tunability response along the (001) BZT-BCT film. The present results suggest the properties of the (001)-oriented film is enhanced owing to the coexistence of orthorhombic and tetragonal phases, which can be attributed to the easing of direct domain switching and polarization rotation due to the low-energy barrier in its distorted state.

## Figures and Tables

**Table 1:** Values of maximum polarization (*P_max_*), remanent polarization (*P_r_*), and coercive field (*E_c_*) for the preferentially epitaxial BZT-BCT films on (001)-, (110)-, and (111)-oriented STO substrates.

Parameters	(001)	(110)	(111)
*P_max_* (μC cm^−2^)	17.88	20.08	18.52
*P_r_* (μC cm^−2^)	~0.84	~1.18	~0.96
*E_c_* (kV cm^−1^)	~31.43	~33.88	~33. 69

**Table 2:** Comparison of energy density and efficiency of different BZT-*x*BCT ceramics. * This work.

Material	Energy Efficiency (*η* %)	Energy Density (J/cm^3^)	Electric Field (kV/cm)	Ref.
[(BaZr_0.2_Ti_0.8_)O_3_]_0.5_[Ba_0.7_Ca_0.3_Ti)O_3_]_0.5_	52	0.10	40	[9]
[(BaZr_0.2_Ti_0.8_)O_3_]_0.5_[Ba_0.7_Ca_0.3_Ti)O_3_]_0.5_	58	78 mJ	25	[11]
[(BaZr_0.1_Ti_0.9_)O_3_]_0.5_[Ba_0.7_Ca_0.3_Ti)O_3_]_0.5_	74	164 mJ	-	[39]
[(BaZr_0.2_Ti_0.8_)O_3_]_0.6_[Ba_0.7_Ca_0.3_Ti)O_3_]_0.4_/ZnO	74	2.61	282	[40]
0.85[(BaZr_0.2_Ti_0.8_)O_3_]_0.6_[Ba_0.7_Ca_0.3_Ti)O_3_]_0.4_-0.15SrTiO_3_	84	0.98	40	[41]
[(BaZr_0.2_Ti_0.8_)O_3_]_0.5_[Ba_0.7_Ca_0.3_Ti)O_3_]_0.5_ (001)	87.62	6.8	1150	*
[(BaZr_0.2_Ti_0.8_)O_3_]_0.5_[Ba_0.7_Ca_0.3_Ti)O_3_]_0.5_ (110)	89.82	8.2	1150	*
[(BaZr_0.2_Ti_0.8_)O_3_]_0.5_[Ba_0.7_Ca_0.3_Ti)O_3_]_0.5_ (111)	90	8.5	1150	*

## Data Availability

The data that support the findings of this study are available from the corresponding authors upon reasonable request.
